# Efficacy of CAD/CAM Glass Fiber Posts for the Restoration of Endodontically Treated Teeth

**DOI:** 10.1155/2022/8621835

**Published:** 2022-01-21

**Authors:** Maira Alejandra Gutiérrez, Camilo Alejandro Guerrero, Paula Alejandra Baldion

**Affiliations:** Departamento de Salud Oral, Facultad de Odontología, Universidad Nacional de Colombia, Bogotá, Colombia

## Abstract

**Objective:**

The aim of this systematic review was to provide an overview of available scientific evidence regarding the comparative efficacy of computer-aided design (CAD) and computer-aided manufacturing (CAM) glass fiber posts with prefabricated and metal cast posts for the restoration of endodontically treated teeth (ETT).

**Methods:**

This systematic review was conducted following the Preferred Reporting Items for Systematic Reviews and Meta-Analyses (PRISMA) guidelines. Electronic and manual searches were performed using the PubMed, SciELO, Cochrane, ScienceDirect, Web of Science, and EBSCO databases. The reference lists of the selected papers were reviewed to identify relevant papers. There were no year restrictions, and eligible studies were those in English publications and describing *in vitro* studies evaluating intraradicular retainers (IRs) for (i) fracture resistance, (ii) bond strength, (iii) adaptation, and (iv) cement layer thickness. Literature reviews, systematic reviews, meta-analyses, case reports*, in vitro* studies with <8 specimens, and noncomparative trials involving prefabricated or metal cast posts were excluded. The authors of this review independently screened the search results, extracted data, and assessed the risk of bias.

**Results:**

No significant differences were found in fracture resistance between prefabricated and CAD/CAM glass fiber posts or between CAD/CAM glass fiber and metal cast posts, although the latter demonstrated higher fracture resistance than the prefabricated glass fiber posts. Restoration with a full crown was not necessary to increase the fracture resistance in the presence of the ferrule effect. CAD/CAM glass fiber and metal cast posts had higher bond strength, lower nanoleakage, and better adaptation to the root canal.

**Conclusions:**

Despite the heterogeneity of methodologies and results reported, the results of these studies indicated that the CAD/CAM glass fiber and metal cast posts showed greater efficacy in terms of fracture resistance, retention, and adaptation, compared to prefabricated glass fiber posts.

## 1. Introduction

The restoration of endodontically treated teeth (ETT) represents a critical problem in restorative dentistry, in which it may be necessary to use intraradicular retainers (IRs) to successfully perform a prosthetic restoration, depending on the specific conditions of the clinical case [1]. There are types of IRs, including fiber-reinforced composite posts, which provide rigidity and resistance to an elastic matrix. Glass fiber posts have a low elastic modulus (40 GPa) compared to other IRs, such as carbon fiber posts (75–215 GPa) and gold cast posts (85 GPa). In IRs with lower elastic moduli, the tooth, cement, and IR undergo deformation at the weakest point, the IR-luting cement-dentin interface, during occlusal function. This in turn causes loss of marginal seal, fracture of the IR or the root, and/or loss of retention [2]. In the most rigid IRs, the forces are distributed along the root, leading to fatigue failure and the possibility of a catastrophic or irrepairable fracture [2]. For this reason, it has been suggested that the materials used to restore an ETT should have physical and mechanical properties similar to those of dentin [3].

Previous studies have stated that glass fiber posts have biomechanical properties similar to those of dentin [4–6], with favorable elastic moduli, fracture resistance, and bond strength, depending on the reinforcement used in the system, and also present optimal esthetic advantages for prosthetic restorations [7]. However, one of the main disadvantages of glass fiber posts is the industrial prefabrication process. Due to the variety of commercial presentations, post diameters have been standardized and may not adequately fit the root canal lumen. This in turn requires further preparation of the canal for the post placement or opting to use a smaller diameter post [8]. Balkenhol et al. [9] determined that the survival rate of posts is primarily related to the adaptation of the post to the anatomy of the root canal, which is a variable of greater relevance than the luting agent itself [10].

With recent advances in technology such as computer-aided design (CAD) and computer-aided manufacturing (CAM), new processes for the manufacture of dental restorations have allowed for the development of a new technique for creating IRs, by creating a digital working model, which allows the scanning, designing, and milling of IRs. Additionally, de Moraes et al. [11] established that the use of CAD/CAM technology eliminates the need to use materials susceptible to dimensional changes. A recent study suggested the use of glass fiber-reinforced polymeric (GFRP) composite blocks for CAD/CAM posts [12]; however, little evidence is available on the efficacy of these compared to conventional IRs such as prefabricated glass fiber and metal cast posts.

Given the multitude of options available, the objective of this systematic review was to provide an overview of the available scientific evidence on the efficacy of CAD/CAM glass fiber posts for the restoration of ETT compared to prefabricated and metal cast posts. In this study, “efficacy” is defined as behavior related to fracture resistance, bond strength, adaptation of the post to the root canal walls, and the thickness of cement layer.

## 2. Methods

This systematic review was carried out according to the parameters defined by the Preferred Reporting Items for Systematic Reviews and Meta-Analysis (PRISMA) guidelines [13]. Based on the population of the study, intervention, comparison, and outcome, the research question was raised, as follows: How effective are CAD/CAM fiberglass posts for ETT restoration compared to prefabricated and metal cast posts? The study was recorded in the database of the International Prospective Registry of Systematic Reviews (Prospero), as number CRD42020215690.

### 2.1. Eligibility Criteria

The criteria for inclusion in the present study were as follows: (1) studies regarding *in vitro* quantitative experiments that evaluated the biomechanical behaviors of IRs, taking into account fracture resistance, post-dentin bond strength, and adaptation of the post to the root canal walls; (2) studies that included samples of human or bovine teeth restored with IRs or those that directly evaluated the materials used in IRs; (3) studies that examined the mechanical properties of IRs through push- or pull-out tests, flexural strength, and/or tensile strength; and (4) studies that microscopically evaluated the adaptation and interfacial nanoleakage of IRs. Literature review studies, systematic reviews, meta-analyses, and case reports were excluded. *In vitro* studies with a sample size <8 specimens were excluded, as were the trials that did not carry out comparative studies including prefabricated or metal cast posts.

### 2.2. Outcomes of Interest

The outcomes of interest in this study were (1) the behavior of the IRs with regard to fracture resistance, (2) the post-cement-dentin bond strength, (3) the adaptation of the post to the root canal walls, and (4) the thickness of the cement layer, specifically comparing glass fiber posts made using a CAD/CAM technique with prefabricated posts and metal cast posts.

### 2.3. Data Sources and Search Strategy

An electronic search was performed using the following databases: PubMed, SciELO, Cochrane, ScienceDirect, Web of Science, EBSCO, and Latin-American and Caribbean System on Health Science Information (LILACS). Additionally, a manual search was performed, in which the reference lists of the included articles were reviewed according to the eligibility criteria. Studies in English and Spanish were selected, there were no restrictions on the year of publication, and the most recent search was performed on February 9, 2021. Keywords and MeSH terms were used, such as “CAD/CAM,” “fiber posts,” “flexural strength,” “fracture resistance,” and “bond strength.” Algorithms were used for the search strategy of this study (Table 1).

### 2.4. Methods of Evaluation and Synthesis of the Included Studies

To assess whether the studies met the inclusion criteria, two authors (C. G. and M. G.) reviewed the titles and abstracts of the articles. Subsequently, based on the inclusion criteria, the abstracts were rereviewed independently by another author (P. B.) to reach a consensus. Next, full-text articles were obtained and reviewed by two authors (C. G. and M. G.), and the final decision was made in consensus with the last author (P. B.). Any disagreement was discussed between the authors (C. G., P. B., and M. G.). The reference lists of the selected articles were then reviewed to verify if there were eligible articles that had been excluded from the electronic search. Finally, full texts of the included studies were evaluated. A protocol for data extraction (PRISMA) was implemented, and the data related to the research question were extracted and recorded in duplicate, using forms designed for this purpose, taking into account (1) citation: location of the study and year of publication, (2) type of study, (3) characteristics of the samples, (4) type of interventions, (5) results obtained, (6) conclusions, and (7) source of financing and conflicts of interest. The data obtained regarding the posts were analyzed, based on the handling of the samples, for (1) fracture resistance, (2) bond strength, (3) adaptation, and (4) cement layer thickness.

### 2.5. Assessment of Risk of Bias and Quality of Included Studies

The methodological quality of the included studies was evaluated as previously described by Sarkis-Onofre et al. [14] and according to the Checklist for Reporting In vitro Studies (CRIS Guidelines) [15]. To assess the quality and risk of bias of the included studies, the following were taken into account: whether or not the allocation of teeth was randomized, whether or not the teeth used were free of caries or restorations, the use of the materials based on the manufacturer's instructions, whether or not the procedures were performed by the same operator, if there was a description of the calculation of the sample size, and the blinding of the test machine operator. The risk of bias for each study was determined by totaling the number of parameters reported, rated as either yes (Y) or no (N). Studies that reported 1-2 parameters were classified as having a high risk of bias, 3-4 as having a medium risk, and 5-6 as having a low risk.

### 2.6. Data Analysis

Data extracted from the included full-text articles were recorded in an evidence table. Variables common to the different articles were identified to facilitate the consolidation of the data from the collection, specimen, sample storage, handling, and processing of the samples, as well as the evaluation techniques and values obtained. These included fracture resistance measured in Newtons (N), bond strength in megapascals (MPa), adaptation of the post to the root canal walls according to the presence of nanoleakage, and the thickness of the cement layer in micrometers (*μ*m), found in the different test groups.

## 3. Results

### 3.1. Search Results

**Figure 1:**
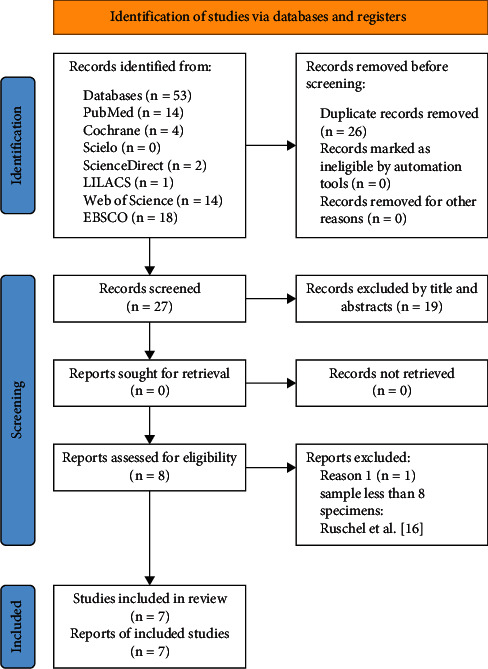
PRISMA flow chart that relates the search information according to the eligibility criteria.

Initially, 53 studies were identified from the different databases, 27 of which were selected after eliminating duplicate reports. Of the 27 studies, 19 were excluded based on title and abstract; therefore, 8 studies were selected for full-text reading, 1 of which was excluded based on the eligibility criteria. The article by Ruschel et al. [16] was excluded because their study included <8 specimens (Figure 1). In total, 7 articles were included in this systematic review (Table 2).

### 3.2. Risk of Bias and Quality Assessment

The risk of bias of the seven included studies was analyzed, and none of which presented a low risk, while five [17, 19, 21–23] presented a moderate risk (71.4%), and two (18, 20) were classified as high risk (28.5%). The bias evaluation was performed according to the parameters listed in Table 3.

### 3.3. Data Analysis and Treatment Effects

The results of the included studies are listed in Table 4 and the comparative efficacy according to the properties studied is analyzed in Table 5. The differences in the methodologies, as shown in Table 2, with respect to the type of tooth, the storage of the samples, the cuts for the laboratory tests, the simulation of the periodontal ligament, the presence or absence of a crown as a final restoration, the instrumentation technique for the filling of the canals, the cementing agent used for the cementation of the IRs, and variations in the characteristics of the laboratory tests evaluated (treatment of the samples, direction of load, surface area of load, crosshead speed, and aging of the samples) showed heterogeneous results, for which only one individual analysis of each *in vitro* study was performed.

### 3.4. Description of Studies and Experimental Models

#### 3.4.1. Study Location

The characteristics of the included studies are summarized in Table 2. Of the included studies, one was carried out in China [17], three were carried out in Lebanon [19, 22, 23], two in Brazil [20, 21], and one was carried out in Italy [18]. Six were conducted in universities [18–23] and one was conducted in a hospital [17]. Only one study was funded by a foundation [20], and two studies reported no funding [19, 22]. Two studies received materials for the execution of the research through dental companies [20, 21], and all studies were published as full text.

#### 3.4.2. Specimens

Human teeth were used in all studies [17–23], five of which used premolars [18–20, 22, 23], one used incisors [17], and one used canine teeth [21]. The sample size was established according to the number of teeth per group of each material to be evaluated. For the studies that evaluated the bond strength (push-out test) [18, 22, 23] and nanoleakage [18], micro samples of 1 mm thick sections were used, and one study used 0.01 mm thick sections to measure the thickness of the cement layer [20].

#### 3.4.3. Storage Protocols

The postextraction teeth used in the tests were stored in different substances. Four studies used 0.5% chloramine T [19, 20, 22, 23], one used water [18], and two did not report the substance used [17, 21]. The storage time of the teeth from extraction to testing ranged from one week to six months. Three studies stored teeth at a temperature of 37°C [18, 20, 21], while the other four studies did not report the storage temperature [17, 19, 22, 23].

#### 3.4.4. Sample Characteristics and Root Canal Preparation Protocols

The samples used in the seven studies reported a root length of 12–15 mm. Any crowns were removed using a rotary cutting instrument with refrigeration, with the amelocemental junction as a reference. One study used a ferrule effect with a 2 mm cut over the amelocemental junction [21]; one study performed this cut at 1 mm [17], and another at 1.5 mm [19]. One study divided the samples into groups with and without a ferrule effect [21], and two studies did not report having performed a coronal cut [22, 23]. Only two studies performed a simulation of the periodontal ligament to assess fracture resistance [17, 21], and five studies did not report such simulations [18–20, 22, 23].

With regard to the techniques for the instrumentation and obturation of root canals, five studies used the ProTaper system (Dentsply Maillefer, Ballaigues, Switzerland) [17, 19, 21–23], one study used the Reciproc and Beefill System (Systat Software, Inc., San José, CA, USA) [18], and one study did not report the technique used [20]. With regard to the root filling technique, three studies used Gates Glidden burs (Dentsply Sirona, York, PA, USA) [19, 22, 23], three used Peeso burs (MANI, Utsunomiya, Japan) [17], one used the White Post bur system (DC; FGM, Joinville, Santa Catarina, Brazil) [21], one study used a diamond bur (KG, Sorensen, SP, Brazil) [20], and one study used size #6 largo burs [18]. After desobturation of the canals to create the IRs, a 4 mm [17] or 5 mm [21] apical seal was used, while the other five studies did not report the length of the seal [18–20, 22, 23]. Only two studies used crowns as restorations over the IRs [17, 20], while the remaining five studies did not report such use [18, 19, 21–23].

#### 3.4.5. Adaptation Techniques of Intraradicular Posts

Pang et al. [17] tested the adaptation of prefabricated glass fiber posts with an indicator to eliminate the blocking points and subsequently performed a sandblasting treatment with alumina. Otherwise, for the metal cast posts, in the work of Tsintsadze et al. [18], the pattern was made with a self-curing acrylic pattern resin (GC, Tokyo, Japan), which was carefully observed by the operator to identify imperfections or bubbles in the material in order to guarantee the adaptation after the casting process. Tsintsadze et al. [18] and Passos et al. [21] evaluated the fit of CAD/CAM glass fiber posts by using polyvinyl siloxane outside the posts. Additionally, Passos et al. [21] polished the surface of the post with finishing and polishing discs (Sof-Lex, 3M ESPE, St. Paul, MN, USA). This technique was repeated until adequate positioning was achieved. Eid et al. [22, 23] reported that CAD/CAM glass fiber posts were adapted in the root canals without the need for adjustments. Likewise, da Costa et al. [20] adjusted the posts for seating with an abrasive bur (Exa-Cerapol, Edenta AG, Heidelberg, Switzerland), while Eid et al. [19] did not report the process for adapting the post to the root canal.

#### 3.4.6. Cementation of Intraradicular Retainers

For the cementation of the IRs, universal self-adhesive cement was used in five studies [17, 19, 21–23], while in one study dual curing cement was used [18], and one study did not report the cementing agent used [20].

#### 3.4.7. Test Methods

*(1) Fracture Resistance*. Of the included studies, four evaluated the fracture resistance of the IRs through compressive strength tests measured in Newtons (N) [17, 19–21]. Eid et al. [19] used a load direction parallel to the longitudinal axis of the tooth, while other studies, such as those of Pang et al. [17] and Passos et al. [21], used a 45° loading direction. da Costa et al. [20] performed a cyclical load to generate fatigue (parallel to the longitudinal axis of the tooth) before the oblique compression test (load direction at 30° until failure). According to the crosshead speed, three studies used a speed of 1 mm/min to apply the load [17, 19, 20], and only one study [21] used a speed of 0.5 mm/min until failure. The reported cycles of the fatigue tests were 300,000 cycles [17] and 1,000,000 cycles [20].

*(2) Bond Strength*. Three studies evaluated the bond strength through push-out tests, measured in megapascals (MPa) [18, 22, 23]. These studies applied the tensile load in the apicocoronal direction in each cut made (1 mm thick sections from the cervical, middle, and apical thirds), placing cylindrical emboli in the center of the posts of each section until failure occurred (extrusion of the section post segment). Two studies [22, 23] performed aging samples employing thermocycling, from 5,000 to 6,000 cycles.

*(3) Interfacial Nanoleakage*. Tsintsadze et al. [18] evaluated the presence of nanoleakages using silver nitrate as a tracer. For the treatment of the samples, the samples were covered with nail varnish, except for the post-dentin-cement interface. They were then observed under a light microscope at 40X magnification for tracer filtration along with the interface (scored from 0 to 4).

*(4) Cement Layer Thickness*. Tsintsadze et al. [18] analyzed six sections (1 mm thick) from each root using a digital microscope to determine the thickness (in *μ*m) of the cement surrounding the posts. da Costa et al. [20] used micro-CT in two specimens from each group (CAD/CAM glass fiber posts and prefabricated glass fiber posts without crowns) using 0.01 mm thick cross sections of the root.

### 3.5. Evaluation of the Efficacy

#### 3.5.1. Fracture Resistance

Of the included studies, four evaluated the fracture resistance of the IRs [17, 19–21]. The most relevant results did not show significant differences in the performance of prefabricated glass fiber and CAD/CAM glass fiber posts [19–21]. No statistically significant differences were found in the fracture resistance of CAD/CAM glass fiber posts and gold cast posts (*p* > 0.05). However, higher fracture resistance values were observed for both materials compared to prefabricated glass fiber posts (*p* < 0.05) [17]. Additionally, the presence of a crown was a determining factor for the fracture resistance of prefabricated glass fiber and CAD/CAM glass fiber posts [20]. Passos et al. [21] did not report any statistically significant differences in the fracture resistance between CAD/CAM glass fiber and prefabricated glass fiber posts, both with and without ferrule (*p* > 0.05). Nevertheless, statistically significant differences were found between samples composed of the same material (CAD/CAM glass fiber post with and without ferrule and prefabricated glass fiber posts with and without ferrule), with increased fracture resistance observed in the samples with ferrule (*p* < 0.05).

#### 3.5.2. Post-Cement-Dentin Bond Strength

Of the studies included, three evaluated the bond strength of IRs [18, 22, 23]. The studies by Eid et al. [22, 23] showed a higher bond strength with CAD/CAM glass fiber posts compared to that obtained with prefabricated glass fiber posts (*p* < 0.001). Similar results were reported by Tsintsadze et al. [18], who found that the group of CAD/CAM glass fiber posts and metal cast posts (base metal alloy Keramit NP, Nobil Metal, Asti, Italy) showed similar bond strength values, which were higher than the bond strength values of the group of prefabricated glass fiber posts. They reported that the post material influenced the bond strength (*p* < 0.05). Eid et al. [22] used petroleum jelly as a lubricant on CAD/CAM glass fiber posts before cementation, which did not affect the bond strength of the metal cast posts.

Tsintsadze et al. [18] reported no differences in the values of bond strength in each root third. In contrast, Eid et al. [23] established that there was a statistically significant difference between the different parts of the root in the CAD/CAM glass (*p*=0.026), CAD/CAM high-density polymer (*p*=0.002), and fiber-reinforced composite (FRC) prefabricated posts (*p*=0.023), since the CAD/CAM glass fiber showed a higher bond strength in the coronal third compared to the middle third (*p*=0.024); meanwhile, with the prefabricated FRC posts, lower values were observed in the coronal third than in the apical third (*p*=0.023). Eid et al. [23] found no influence of thermal aging of the samples on the bond strength values in any of the groups (*p*=0.536).

#### 3.5.3. Adapting the Post to the Root Canal Walls

Tsintsadze et al. [18] evaluated the internal adaptation of IRs by employing an interfacial nanoleakage test. Statistical analysis showed that the lowest score was observed in the base metal alloy cast posts, compared to CAD/CAM glass fiber and prefabricated glass fiber posts. However, the latter two groups showed similar values.

#### 3.5.4. Thickness of Cement Layer

The studies by Tsintsadze et al. [18] and da Costa et al. [20] showed a statistically significant difference in cement thickness based on the type of post and the root thirds evaluated. Tsintsadze et al. [18] reported that the lowest cement thickness was found in the metal cast posts, followed by CAD/CAM posts, and finally by prefabricated glass fiber posts. Similar results were reported by da Costa et al. [20], who found a greater luting agent thickness in prefabricated glass fiber posts compared to CAD/CAM glass fiber posts. According to the category of voids present in the cement film, CAD/CAM glass fiber posts had a higher percentage (80%) of cases in which voids were absent in all root thirds.

## 4. Discussion

This systematic review aimed to compile and analyze the available scientific evidence regarding the efficacy of glass fiber posts made using CAD/CAM techniques compared to prefabricated posts and metal cast posts.

### 4.1. Summary of Main Results

No statistically significant differences were observed in IR fracture resistance between prefabricated glass fiber and CAD/CAM glass fiber posts [19–21]. Similarly, there was no evidence of a difference between the fracture resistances of the CAD/CAM glass fiber and the metal cast posts; however, the latter demonstrated a better fracture resistance than prefabricated glass fiber posts [17]. The presence of a crown was not a determining factor for fracture resistance [20], but the presence of a ferrule increased fracture resistance [21]. With respect to the post-cement-dentin bond strength, the IR material was found to influence bonding values [18]. Prefabricated glass fiber posts have lower bond strength than CAD/CAM glass fiber posts [18, 22, 23] and metal cast posts [18]. Additionally, CAD/CAM glass fiber posts showed greater bond strength in the coronal than in the middle third, while the prefabricated FRC posts showed a lower bond strength in the coronal than in the apical third. The influence of thermal aging on bond strength values was not observed [23]. The metal cast posts demonstrated better internal adaptation than CAD/CAM glass fiber and prefabricated glass fiber posts, which demonstrated a more significant mismatch to the root canal walls [18].

### 4.2. Quality of the Evidence, Limitations, and Possible Biases in the Review

The results of the studies included in the present review correspond to *in vitro* studies, which must be interpreted with caution, since they cannot wholly reflect all circumstances of a clinical situation. Additionally, the differences in the type of tooth, storage of the samples, instrumentation techniques for the sealing and desobturation of root canals, cement agent, shape of the root cuts for the laboratory tests, and variations of the characteristics of the laboratory tests (treatment of samples, type of load applied, cross-sectional area, crosshead speed, and aging samples) led to heterogeneous results, whereby only an individual analysis of the studies was conducted. According to the bias evaluation, none of the studies showed a low risk of bias (Table 3), which corresponds to a 100% risk of medium and high bias (Table 3).

### 4.3. Agreements and Disagreements with Other Studies or Reviews

The results of the studies included in the present review showed that the biomechanical behaviors of the different types of IRs are controversial, since some authors [19–21] found no significant differences in fracture resistance between the two types of posts and metal cast posts [19–21]. However, in contrast to these results, Pang et al. [17] reported that the CAD/CAM glass fiber posts and metal cast posts showed higher fracture resistance when compared to prefabricated glass fiber posts [17]. In contrast, Torres et al. [24] established that glass fiber posts increased ETT fracture resistance compared to gold cast posts, suggesting that multiple additional factors influence the biomechanical behaviors of the restored ETT with IRs.

An essential factor for the success of the restoration of the ETT is the material from which the IR is composed. Authors such as Falcão et al. [25] evaluated different materials for creating CAD/CAM IRs, such as nanoceramic resin composite post, Lava Ultimate (3M ESPE, St. Paul, MN, USA), and posts of hybrid ceramic, Vita Enamic (Vita, Bad Säckingen, Germany), which demonstrated good performance in terms of fracture resistance as a viable alternative to restore on oval-shaped roots and with esthetic commitment. According to Belli et al. [3], the elastic modulus of the material used for IRs influences the fracture resistance because a post and core with a high elastic modulus generate a greater concentration of stresses on the root dentin, resulting in an increased risk of fracture of the IR-tooth system. Therefore, the preceding study suggested that a post and core system using a material with an elastic modulus similar to that of dentin should be implemented to ensure adequate stress dissipation and thus avoid areas of concentrated stress in the walls of the root that can generate failures at the post-cement-dentin interface or radicular fractures [3, 26]. From this perspective, glass fiber systems have shown excellent biomechanical behavior compared to other materials [27]. The fibers have high tensile strength [28] and provide rigidity and resistance to a matrix that in turn is elastic; meanwhile, the metal cast post, being more rigid, transmits the stress directly to the remaining dental structure, leading to fatigue failures [2].

The above description becomes evident when analyzing the type of failure. Prefabricated glass fiber and gold cast posts were found to have catastrophic fractures in all cases, while CAD/CAM glass fiber posts presented repairable fractures in six cases and irrepairable fractures in four cases [17]. These results agree with those reported by Figueiredo et al. [29], who found catastrophic failures in ETT restored with metal cast and glass fiber posts, with similar failure characteristics in both groups [29]. Additionally, Torres et al. [24] identified that the most frequent fracture site with gold cast posts was in the middle third (47.7%), which corresponds to a type of irrepairable failure, while the most frequent site with glass fiber posts was the cervical third (42.9%), which was thought to be a repairable fracture [24]. In contrast, Eid et al. [19] reported no catastrophic failures using prefabricated glass fiber, CAD/CAM glass fiber, or hybrid ceramic CAD/CAM posts [19].

The analysis of the differential behavior of the failure of the different IR systems is based on the biomechanical behavior of the structures that make up the system and the interfaces formed within. Regarding the failure analysis, Marchionatti et al. [30] concluded that failures of fiber posts were due to the loss of retention, while the metal cast posts showed fractures of the root or the post, as well as post decementation [30]. The above shows the number of possible types of failure in metal cast posts, rigid systems that generate a flexural deformation of the root under occlusal stresses, which do not produce a synchronous deformation of the metal cast post with the root tissue, thus creating concentrated stress zones that exceed the maximum stress values up to the point of ultimate strength where the fracture occurs [31]. The opposite is true of CAD/CAM glass fiber posts, which have an elastic modulus similar to dentin, meaning they adapt well to the root canal and optimize the resistance of the post and core system, suggesting that a CAD/CAM glass fiber post reduces the cement thickness at the bond interface and provides a better distribution of stress. Therefore, both the bond strength and fracture resistance of the CAD/CAM glass fiber posts were higher than those of the other types of IRs [32].

Regarding the influence on the fracture resistance and the amount of coronal tissue remaining to generate a ferrule effect in ETT, Passos et al. [21] established that the presence of a ferrule increased the fracture resistance values in restorations made up of the same type of material [21]. This is in agreeance with the study by Marchionatti et al. [30], who concluded that the presence of dental remnants and the ferrule increased ETT survival [30] due to an increase in fracture resistance [33]. Additionally, it has been described that the ferrule effect allows a better distribution of stress towards the dental root [34].

Another crucial factor to consider is the change in the behavior of the fracture resistance of ETT when they are restored with a full crown. Catastrophic fractures were found in teeth restored with CAD/CAM glass fiber posts without a prosthetic crown [20], which may reinforce the restoration system by dissipating oblique stresses [35]. However, the results of da Costa et al. [20] showed that the presence or absence of a prosthetic crown does not influence fracture resistance [20], which highlights the importance of the conformation of the root portion more so than the coronal one in this type of restoration.

Regarding the post-cement-dentin bond strength, the adaptation of the IR to the canal anatomy influenced the bond strength values [18]. Studies have reported greater bond strength with CAD/CAM glass fiber posts than with prefabricated glass fiber posts, with which the thickness of the cement layer is increased [20, 22] and adaptation to the root canal walls is decreased [18, 22, 23]. Additionally, the relationship of an adequate adaptation of the IR with lower nanoleakage [20] and an excellent stabilization of the bonding interface is evident. This interface can be affected by other factors such as moisture, the highly organic nature of the root dentin rich in dentinal tubules, the sensitivity of the bonding technique used, the type of cementing agent, the degree of conversion obtained [36], and polymerization shrinkage of resin cement [37]. Skupien et al. [38] reported that certain types of cementing agents, such as self-adhesive resin cement, are more sensitive to the technique used to perform cementation than conventional resin cement [38]. Advantages such as the presence of a thinner layer of cementing agent [39] which decreases the percentage of polymerization shrinkage [40] and the possibility of bubble and void formation can be definitive to increase the post-cement-dentin bond strength [41]. The adaptation of CAD/CAM fiber and cast posts plays a predominant role in the success of ETT.

Regarding the passive settlement of IRs into the root canal, Eid et al. [22] reported that CAD/CAM glass fiber posts have the advantage of seating without the need for adjustments. It is possible that establishing a detailed copy in the design software of the space required for the luting cement confers a precise adaptation of the posts to the canal [22, 23]. Fransson et al. [42] considered an adaptation discrepancy of up to 150 *μ*m acceptable [42, 43]. Additionally, the standard of the International Organization for Standardization (ISO) 4049 of the year 2019 established an ideal cement layer thickness of 10–50 *μ*m [44]. However, the studies included in the present review implemented an 80 *μ*m cement thickness in the design software [22, 23], based on the accepted discrepancy values in the literature.

### 4.4. Implications for Practice

With the possible limitations of the present systematic review, taking into account the heterogeneity of methodologies and reported results, the analyses indicated that the CAD/CAM glass fiber posts had greater efficacy in terms of bond strength and post adaptation compared to prefabricated glass fiber posts and results similar to those of metal cast posts. However, it was evident that the biomechanical behaviors of the different types of IRs are still controversial because, for fracture resistance, in some studies, no significant differences were observed between the two types of posts and the metal cast post. However, other authors reported that CAD/CAM glass fiber and metal cast posts showed higher fracture resistance values when compared to prefabricated glass fiber posts. Therefore, a comprehensive analysis of the multiple factors that can influence the biomechanical behaviors of IR-restored ETT is recommended.

### 4.5. Implications for Future Research

Further investigations, such as *in vivo* studies, are needed to determine the efficacy of CAD/CAM glass fiber posts, since the currently available literature evaluated the biomechanical behaviors in *in vitro* studies, which, due to their experimental nature, do not allow an adequate correlation of the performance of the posts under clinical circumstances.

## 5. Conclusions

CAD/CAM posts demonstrated positive behaviors in terms of retention, fracture resistance, bond strength, and adaptation compared to the other types of IRs. Although long-term clinical trials are needed to corroborate the usefulness of this alternative, CAD/CAM glass fiber posts may be considered an effective alternative for ETT restoration.

## Figures and Tables

**Table 1:** Search algorithms in the different databases.

Data base	Search algorithm	Limiters applied
PubMed	CAD/CAM OR CAD-CAM ((fiber posts [Title/Abstract])) AND (flexural strength [Title/Abstract] OR fracture resistance [Title/Abstract] OR bond strength [Title/Abstract]) NOT (finite element analysis [Title/Abstract] OR clinical study [Title/Abstract] OR review [Title/Abstract])	Language: English
Cochrane	(“CAD/CAM” OR “CAD-CAM”) AND (fiber posts) AND (flexural strength OR fracture resistance OR bond strength) NOT (finite element analysis OR clinical study OR review)	Title, abstract, keyword
SciELO	(ab:(CAD/CAM OR CAD-CAM)) AND (ab:(fiber posts)) AND (ab:(flexural strength OR fracture resistance OR bond strength)) NOT (ab:(finite element analysis OR clinical Study OR review))	Abstract
ScienceDirect	(“CAD/CAM” OR “CAD-CAM”) AND (fiber posts) AND (flexural strength OR fracture resistance OR bond strength) NOT (finite element analysis OR clinical Study OR review)	Subject area: medicine and dentistry Article type: research article
LILACS	(CAD/CAM OR CAD-CAM) AND (fiber posts) AND (flexural strength OR fracture resistance OR bond strength) AND NOT (finite element analysis OR clinical Study OR review)	Title, abstract, topic Language: English
Web of Science	(AB = ((“CAD/CAM” OR “CAD-CAM”) AND (fiber posts) AND (flexural strength OR fracture resistance OR bond strength) NOT (finite element analysis OR clinical Study OR review)))	Main collection of Web of Science advanced search Language: English
EBSCO	(“CAD/CAM” OR “CAD-CAM”) AND (fiber posts) AND (flexural strength OR fracture resistance OR bond strength) NOT (finite element analysis OR clinical Study OR review)	MEDLINE Complete

**Table 2:** Characteristics of the seven studies included in the revision.

Study	Specimen	Sample storage	Sample characteristics	Sample preparation	Intraradicular post cementation	Intraradicular posts
Reference	Type of tooth/species/sample size	Substance/time/T°	Radicular length/coronal section/apical seal	Periodontal ligament simulation/instrumentation technique/crown presence	Luting agent	Studied groups
Pang et al. (2019) [17]	Upper central incisors/human/enlarged root canals N = 30 (teeth) *n* = 10	Not reported	13 mm/1 mm above CEJ/4 mm	YES/Manual ProTaper until F3, Crown Down, Peeso reamer #2/YES	RelyX Unicem 2 (3 M ESPE). Self-adhesive universal cement	Group 1 (A): CAD/CAM GFP Group 2 (B): prefabricated GFP Group 3 (C): CP gold alloy
Tsintsadze et al. (2017) [18]	Uniradicular premolars/human/ N = 30 (teeth) *n* = 10 n = 6 (push-out) *n* = 4 (nanofiltration) SEM (representative sections per group)	Water/ 1 week/ 37°C	Not reported	Not reported/Reciproc and Beefill 2 in 1 system, Largo reamer #6/Not reported	Gradia Core (GC). Dual cure cement	Group 1: prefabricated GFP Group 2: CP Group 3: CAD/CAM GFP
Eid et al. (2019) [19]	Mandibular second premolars/human/ N = 30 *n* = 10	Chloramine-T 0.5%/ 2 months/ Not reported	12 to 14 mm 1.5 mm above CEJ/ Not reported	Not reported/ProTaper nickel-titanium rotary system, Gates Glidden drill #1, #2, #3/Not reported	RelyX U200 (3 M ESPE). Self-adhesive cement	Group 1 (RXP): prefabricated GFP Group 2 (BLC): CAD/CAM GFP Group 3 (AMC): CAD/CAM hybrid ceramic disc posts
da Costa et al. (2017) [20]	Uniradicular premolars/ human/ N = 40 n = 10 n = 2 (micro-CT de CPn y PPn)	Initial storing: Chloramine-T 0.5%/ 6 months/ Not reported Final storing: incubator at 37°C-100% humidity	Not reported	Not reported/Diamond bur/YES	Not reported	Group 1 (PPc): prefabricated GFP with crown Grupo 2 (PPn): prefabricated GFP without crown Grupo 3 (CPc): CAD/CAM GFP with crown Grupo 4 (CPn): CAD/CAM GFP without crown
Passos et al. (2017) [21]	Mandibular canines/human/ N = 40 n = 10	Not reported/ 24 h/ 37°C	15 mm/without ferrule (*n* = 20) 2 mm (*n* = 20)/5 mm	YES/ProTaper Universal files, Whitepost DC posts #0.5, 1, 2, 3/Not reported	RelyX U200 (3 M ESPE). Self-adhesive cement	Group 1 (VE): CAD/CAM GFP without ferrule Group 2 (VEF): CAD/CAM GFP with ferrule Group 3 (WP): prefabricated GFP^*∗*^ without ferrule Group 4 (WPF): prefabricated GFP^*∗*^ with ferrule
Eid et al. (2019) [22]	Mandibular uniradicular premolars/human/ N = 30 n = 10	Chloramine-T 0.5%/ 2 months/ Not reported	14 mm/Not reported/Not reported	Not reported/ProTaper nickel-titanium rotary system, Gates Glidden drill #2, Peeso reamer #1–3 (gradually)/Not reported	RelyX U200 (3 M ESPE). Self-adhesive cement	Group 1 (CP): CAD/CAM FRRP Group 2 (CPL): CAD/CAM FRRP^*∗*^ lubricated with Vaseline Group 3 (RXP): prefabricated GFP
Eid et al. (2019) [23]	Mandibular uniradicular premolars/human/ N = 80 n = 20	Chloramine-T 0.5%/ 2 months/ Not reported	14 mm/ Not reported/Not reported	Not reported/ProTaper nickel-titanium rotary system, Gates Glidden drill #2, Peeso reamer #1–3 (gradually)/Not reported	RelyX U200 (3 M ESPE). Self-adhesive cement	Group 1 (BLC): CAD/CAM GFP Group 2 (AMC): high-density polymer CAD/CAM posts Group 3 (BLP): prefabricated FRRP Group 4 (RXP): prefabricated GFP

CEJ: cementoenamel junction; FRRP: fiber-reinforced resin posts; GFP: glass-fiber posts; CP: cast posts; RT: room temperature.

**Table 3:** Risk of bias according to the information evaluated from the materials and methods of the selected studies.

Study	Randomization^*∗*^	Teeth used ^*∗∗*^	Manufacturer's instructions ^*∗∗∗*^	Calibrated operator^ꝉ^	Sample size^§^	Operator blinding	Risk of bias
Pang et al. [17]	Y	Y	Y	Y	N	N	Moderate
Tsintsadze et al. [18]	Y	N	Y	N	N	N	High
Eid et al. [19]	Y	Y	Y	Y	N	N	Moderate
da Costa et al. [20]	Y	N	Y	N	N	N	High
Passos et al. [21]	Y	Y	Y	Y	N	N	Moderate
Eid et al. [22]	N	Y	Y	Y	N	N	Moderate
Eid et al. [23]	Y	Y	Y	Y	N	N	Moderate

^*∗*^Random assignment of teeth to test groups. ^*∗∗*^Teeth free of cavities or restorations. ^*∗∗∗*^Procedures performed according to manufacturer's instructions. ^ꝉ^Procedures carried out by the same operator. ^§^Sample size calculation.

**Table 4:** Summary of the results of the 7 included studies.

Study	Studied properties/	Unit of measurement	Studied groups	Conclusions
No.	Laboratory tests		Results by properties	
[17]	Fracture resistance/fatigue and static loading Failure mode/observational	N	Mean fracture resistance: Group 1 (A): (927,6 ± 275,6) Group 2 (B): (616,5 ± 154,9) Group 3 (C): (967,9 ± 157,5) Group 1 and Group 3: (*p* > 0.05) Group 2: (*p* < 0.05, Table 1). Group 1 (A): 6 cases of repairable fracture, 4 cases of irrepairable fracture Group 2 (B): 7 cases of irrepairable fracture Group 3 (C): 9 cases of irrepairable fracture	Integrated CAD/CAM restoration of glass fiber post and core for widened root canals can increase the fracture resistance of the root and reduce the occurrence of irrepairable root fractures.
[18]	Bond strength/*push-out* test Cement layer thickness/SEM^*∗*^ Nanoleakage/interfacial nanoleakage in AgNO_3_	MPa *μ*m	Bond strength: Group 1: (8,19 ± 3,62) Group 2: (26,41 ± 18,77) Group 3: (17,12 ± 7,73) Cement thickness: Group 1: (654 ± 22,5) Group 2: (106 ± 53) Group 3: (162 ± 24) Average nanoleakage values: Group 1: 4 (>75% with nanoleakage) Group 2: 1 (25% of the interface shows nanoleakage) Group 3: 3 (50% to <75% with nanoleakage)	CAD/CAM GFP^*∗*^ could represent a valid alternative to posts traditionally used in the restoration of endodontically treated teeth with oval or wide root canals, offering the advantages of better esthetics, retention, and cement thickness values that are comparable to cast posts.
[19]	Fracture resistance Failure mode/SEM	N	Fracture resistance: Group 1 (RXP): (426.08 ± 128.26) Group 2 (BLC): (367.06 ± 72.34) Group 3 (AMC): (620.02 ± 54.29) Group 1: mixed failure Groups 2 and 3: cohesive failures with no catastrophic failures reported in all groups.	The one-piece post and core can be successfully milled from FRR blocks and high-density polymer material discs using CAD/CAM technology. High-density polymer CAD/CAM GFP^*∗*^ showed a better performance than prefabricated fiber posts.
[20]	Cement layer thickness/micro-CT^*∗*^ Fracture resistance/fatigue testing Failure mode/observational	*μ*m N	Group 2 (PPn): Cervical: 220.5(76.7) Middle: 204.2(66.5) Apical: 180.1(64.7) Group 4 (CPn): Cervical: 121.0(45.1) Middle: 121.5(29.7) Apical: 112.3(35.6) Fracture resistance: Group 1 (PPc): 716.9 (260.8) Group 2 (PPn): 640.4 (171.9) Group 3 (CPc): 778.0 (232.5) Group 4 (CPn): 792.9 (265.3) Groups 1 and 3 did not show any visible damage (type 0). Type 4 (catastrophic) fractures occurred in Groups 4 and 1.	CAD/CAM GFP^*∗*^ do not affect the fracture resistance of widened root canals or cause catastrophic root failure when the tooth is rehabilitated with zirconia crowns.
[21]	Fracture resistance/fracture testing Failure mode/optical microscope	N	No statistically significant difference was found in fracture resistance under oblique loading in the case of hybrid CAD/CAM blocks and fiber posts were used, in both the ferrule and no-ferrule groups. The failure mode distribution of the group without ferrule effect did not present unfavorable failures, while the failures in the ferrule groups were distributed between the favorable and unfavorable groups.	Hybrid CAD/CAM blocks can be considered as an alternative restoration system for post and core restorations. More clinical and laboratory research needs to be done to support the improvement of this system.
[22]	Bond strength/push-out test Failure mode/stereo microscope	MPa	Bond strength was significantly lower in Group 3 (RXP) (8.54 ± 3.35 MPa) compared to Group 1 (CP) (12.10 ± 1.38 MPa), while no significant differences were found between the other groups. The failure was mainly adhesive for Groups 2 and 3 and adhesive and mixed for Group 1.	The use of CAD/CAM custom FRRP^*∗*^ has a positive effect on bond strength to root canal walls compared to prefabricated GFP^*∗*^. Self-adhesive resin cements to radicular dentin did not significantly improve the bond strength of prefabricated posts, where friction appears to play a predominant role in post retention.
[23]	Bond strength/micro-CT^*∗*^ Failure mode/stereo microscope	MPa	Bond strength: *CAD/CAM GFP*^*∗*^*groups* Group 1 (BLC): (12.43 ± 2.15) Group 2 (AMC): (10,65 ± 1,77) *Prefabricated GFP*^*∗*^*groups* Group 3 (BLP): (9,67 ± 2,98) Group 4 (RXP): (8,91 ± 3,09) The failures were adhesive between the cement and dentin for all groups except Group 2, where an adhesive failure was observed between the cement and the post.	CAD/CAM manufacturing technology improved post retention in the root canal and enabled a complete digital workflow compared to GFP^*∗*^. Fiber-reinforced composites performed better than high-density polymers in terms of resistance to adhesive failure between post and cement. Aging did not affect the bond strength of GFP^*∗*^ and CAD/CAM GFP^*∗*^ to radicular dentin.

N: Newtons; *μ*m: micrometers; MPa: MegaPascals; SEM: scanning electron microscopy; micro-CT: computed microtomography; FRRP: fiber-reinforced resin posts; GFP: glass fiber posts; CP: cast posts.

**Table 5:** Results of the 7 included studies according to the groups studied and their comparative efficacy.

Study	Intraradicular posts	Studied properties			
Reference	Studied groups	*Fracture resistance*	*Bond strength*	*Nanoleakage*	*Cement layer thickness*
Pang et al. [17]	GFP^*∗*^ GFP CAD/CAM CP	- = =	-----	-----	-----
Tsintsadze et al. [18]	GFP GFP CAD/CAM CP	-----	- + ++	++ + -	+ = =
Eid et al. [19]	GFP GFP CAD/CAM	= =	-----	-----	-----
da Costa et al. [20]	GFP GFP CAD/CAM	= =	-----	-----	+ -
Passos et al. [21]	GFP GFP CAD/CAM	= =	-----	-----	-----
Eid et al. [22]	GFP GFP CAD/CAM	-----	- +	-----	-----
Eid et al. [23]	GFP GFP CAD/CAM	-----	- +	-----	-----

GFP: glass fiber posts. CP: cast posts. +: greater efficacy. –: less efficacy. =: equal efficacy. -----: property not evaluated.

## References

[1] Duc O., Krejci I. (2009). Effects of adhesive composite core systems on adaptation of adhesive post and cores under load. *Journal of Dentistry*.

[2] Torbjörner A., Fransson B. (2004). A literature review on the prosthetic treatment of structurally compromised teeth. *The International Journal of Prosthodontics*.

[3] Liu P., Deng X.-L., Wang X.-Z. (2010). Use of a CAD/CAM-fabricated glass fiber post and core to restore fractured anterior teeth: a clinical report. *The Journal of Prosthetic Dentistry*.

[4] Von Krammer R. (1996). A time-saving method for indirect fabrication of cast posts and cores. *The Journal of Prosthetic Dentistry*.

[5] Streacker A. B., Kenyon B. (2005). A simplified technique for fabricating an acrylic resin cast dowel and core pattern. *The Journal of Prosthetic Dentistry*.

[6] Memari Y., Mohajerfar M., Armin A., Kamalian F., Rezayani V., Beyabanaki E. (2019). Marginal adaptation of CAD/CAM all-ceramic crowns made by different impression methods: a literature review. *Journal of Prosthodontics*.

[7] Owen T. A., Barber M. (2018). Direct or indirect post crowns to restore compromised teeth: a review of the literature. *British Dental Journal*.

[8] Tey K. C., Lui J. L. (2014). The effect of glass fiber-reinforced epoxy resin dowel diameter on the fracture resistance of endodontically treated teeth. *Journal of Prosthodontics*.

[9] Balkenhol M., Wöstmann B., Rein C., Ferger P. (2007). Survival time of cast post and cores: a 10-year retrospective study. *Journal of Dentistry*.

[10] Racanshad S. (2003). An in vitro study of coronal microlealtage in endodontically-treated teeth restored with posts. *Australian Endodontic Journal*.

[11] de Moraes A. P., Poletto Neto V., Boscato N., Pereira-Cenci T. (2016). Randomized clinical trial of the influence of impression technique on the fabrication of cast metal posts. *The Journal of Prosthetic Dentistry*.

[12] Sary S. B., Samah M. S., Walid A. A. Z. (2019). Effect of restoration technique on resistance to fracture of endodontically treated anterior teeth with flared root canals. *J Biomed Res*.

[13] Moher D., Liberati A., Tetzlaff J., Altman D. G., Group T. P. (2009). Preferred reporting Items for systematic reviews and meta-analyses: the PRISMA statement. *PLoS Medicine*.

[14] Sarkis-Onofre R., Jacinto R. d. C., Boscato N., Cenci M. S., Pereira-Cenci T. (2014). Cast metal vs. glass fibre posts: a randomized controlled trial with up to 3 years of follow up. *Journal of Dentistry*.

[15] Krithikadatta J., Datta M., Gopikrishna V. (2014). CRIS Guidelines (Checklist for Reporting In-vitro Studies): a concept note on the need for standardized guidelines for improving quality and transparency in reporting in-vitro studies in experimental dental research. *Journal of Conservative Dentistry*.

[16] Ruschel G. H., Gomes É. A., Silva-Sousa Y. T. (2018). Mechanical properties and superficial characterization of a milled CAD-CAM glass fiber post. *Journal of the Mechanical Behavior of Biomedical Materials*.

[17] Pang J., Feng C., Zhu X. (2019). Fracture behaviors of maxillary central incisors with flared root canals restored with CAD/CAM integrated glass fiber post-and-core. *Dental Materials Journal*.

[18] Tsintsadze N., Juloski J., Carrabba M. (2017). Performance of CAD/CAM fabricated fiber posts in oval-shaped root canals: an in vitro study. *American Journal of Dentistry*.

[19] Eid R., Juloski J., Ounsi H., Silwaidi M., Ferrari M., Salameh Z. (2019). Fracture resistance and failure pattern of endodontically treated teeth restored with computer-aided design/computer-aided manufacturing post and cores: a pilot study. *The Journal of Contemporary Dental Practice*.

[20] da Costa R. G., Freire A., Caregnatto de Morais E. C., Machado de Souza E., Correr G. M., Rached R. N. (2017). Effect of CAD/CAM glass fiber post-core on cement micromorphology and fracture resistance of endodontically treated roots. *American Journal of Dentistry*.

[21] Passos L., Barino B., Laxe L., Street A. (2017). Fracture resistance of single-rooted pulpless teeth using hybrid CAD/CAM blocks for post and core restoration. *International Journal of Computerized Dentistry*.

[22] Eid R., Azzam K., Ounsi H. (2019). Influence of adaptation and adhesion on the retention of computer-aided design/computer-aided manufacturing glass fiber posts to root canal. *The Journal of Contemporary Dental Practice*.

[23] Eid R. Y., Koken S., Baba N. Z., Ounsi H., Ferrari M., Salameh Z. (2019). Effect of fabrication technique and thermal cycling on the bond strength of cad/cam milled custom fit anatomical post and cores: an in vitro study. *Journal of Prosthodontics*.

[24] Torres-Sánchez C., Montoya-salazar V., Córdoba P. (2013). Fracture resistance of endodontically treated teeth restored with glass fiber reinforced posts and cast gold post and cores cemented with three cements. *The Journal of Prosthetic Dentistry*.

[25] Falcão Spina D. R., Goulart da Costa R., Farias I. C., da Cunha L. G., Ritter A. V., Gonzaga C. C. (2017). CAD/CAM post-and-core using different esthetic materials: fracture resistance and bond strengths. *American Journal of Dentistry*.

[26] Belli S., Eraslan O., Eraslan O., Eskitascioglu M., Eskitascioglu G. (2014). Effects of NaOCl, EDTA and MTAD when applied to dentine on stress distribution in post-restored roots with flared canals. *International Endodontic Journal*.

[27] Aggarwal V., Singla M., Miglani S., Kohli S. (2012). Comparative evaluation of fracture resistance of structurally compromised canals restored with different dowel methods. *Journal of Prosthodontics*.

[28] Lamichhane A., Xu C., qiang F.-q. (2014). Dental fiber-post resin base material: a review. *The Journal of Advanced Prosthodontics*.

[29] Figueiredo F. E. D., Martins-Filho P. R. S., Faria-E-Silva A. L. (2015). Do metal post-retained restorations result in more root fractures than fiber post-retained restorations? A systematic review and meta-analysis. *Journal of Endodontics*.

[30] Marchionatti A. M. E., Wandscher V. F., Rippe M. P., Kaizer O. B., Valandro L. F. (2017). Clinical performance and failure modes of pulpless teeth restored with posts: a systematic review. *Brazilian Oral Research*.

[31] Sobek J., Veselý V., Seitl S. (2014). Combination of wedge splitting and bending fracture test-crack tip stress field and nonlinear zone extent analysis. *Advanced Materials Research*.

[32] Borzangy S. S., Saker S. M., Al-Zordk W. A. (2019). Effect of restoration technique on resistance to fracture of endodontically treated anterior teeth with flared root canals. *J Biomed Res*.

[33] Jiangkongkho P., Kamonkhantikul K., Takahashi H., Arksornnukit M. (2013). Fracture resistance of endodontically treated teeth using fiber post with an elastic modulus similar to dentin. *Dental Materials Journal*.

[34] Santos-Filho P. C. F., Veríssimo C., Soares P. V., Saltarelo R. C., Soares C. J., Marcondes Martins L. R. (2014). Influence of ferrule, post system, and length on biomechanical behavior of endodontically treated anterior teeth. *Journal of Endodontics*.

[35] Belli S., Kalkan M. (2002). Evaluation of two post core systems using two different methods (fracture strength test and a finite elemental stress analysis). *Journal of Endodontics*.

[36] Bitter K., Paris S., Pfuertner C., Neumann K., Kielbassa A. M., Bitter K. (2009). Morphological and bond strength evaluation of different resin cements to root dentin. *European Journal of Oral Sciences*.

[37] Yumi T., Suzuki U., Gomes-filho J. E., Gallego J. (2009). Mechanical properties of components of the bonding interface in different regions of radicular dentin surfaces. *The Journal of Prosthetic Dentistry*.

[38] Skupien J. A., Sarkis-Onofre R., Cenci M. S., Moraes R. R. d., Pereira-Cenci T. (2015). A systematic review of factors associated with the retention of glass fiber posts. *Brazilian Oral Research*.

[39] Moustapha G., Alshwaimi E., Silwadi M., Ounsi H., Ferrari M., Salameh Z. (2019). Marginal and internal fit of CAD/CAM fiber post and cores. *International Journal of Computerized Dentistry*.

[40] Grandini S., Goracci C., Monticelli F., Borracchini A., Ferrari M. (2005). SEM evaluation of the cement layer thickness after luting two different posts. *The Journal of Adhesive Dentistry*.

[41] D’Arcangelo C., Cinelli M., De Angelis F., D’Amario M., Aquila L. (2007). The effect of resin cement film thickness on the pullout strength of a fiber-reinforced post system. *The Journal of Prosthetic Dentistry*.

[42] Fransson B., Øilo G., Gjeitanger R. (1985). The fit of metal-ceramic crowns, a clinical study. *Dental Materials*.

[43] Syrek A., Reich G., Ranftl D., Klein C., Cerny B., Brodesser J. (2010). Clinical evaluation of all-ceramic crowns fabricated from intraoral digital impressions based on the principle of active wavefront sampling. *Journal of Dentistry*.

[44] https://www.iso.org/standard/67596.html.

